# Persistent primitive hypoglossal artery: a classification-based approach to understanding and managing a rare vascular anomaly

**DOI:** 10.1007/s00701-025-06654-w

**Published:** 2025-10-23

**Authors:** Nshaat Abdrabou Elsayed, Ahmed Azhar Ali

**Affiliations:** Department of Vascular Surgery, Cardiothoracic and Vascular Surgery Center (CVSC), University Hospital, Mansoura University, Mansoura, 35516 Egypt

**Keywords:** Persistent primitive hypoglossal artery, PPHA, Carotid-basilar anastomosis, Vascular anomalies, Cerebrovascular disease, Classification, Carotid endarterectomy

## Abstract

**Background:**

Persistent primitive hypoglossal artery (PPHA) is a rare embryological variant of the cerebral circulation in which the posterior circulation is supplied through a persistent embryonic carotid–basilar anastomosis. While often discovered incidentally, PPHA may have important clinical consequences when associated with atherosclerotic disease, aneurysms, or other cerebrovascular abnormalities.

**Objective:**

To introduce a structured clinical classification system for PPHA that captures its anatomical spectrum, pathological associations, and symptomatic presentations, with the aim of improving diagnostic precision and procedural planning.

**Methods:**

A six-category framework was developed to stratify PPHA according to its clinical and radiological features. This classification emphasizes anatomical distinctiveness and pathological relevance to facilitate risk stratification and therapeutic decision-making.

**Results:**

The proposed classification defines six categories: (1) isolated asymptomatic PPHA, (2) PPHA with stenosis, (3) aneurysmal PPHA, (4) PPHA with carotid artery stenosis, (5) PPHA with cranial nerve compression, and (6) PPHA associated with other vascular anomalies. Organizing PPHA into these categories provides a practical system for clinical assessment and intervention planning.

**Conclusion:**

This novel classification addresses the current absence of a standardized clinical framework for PPHA. Its adoption may enhance physician awareness, improve patient safety during cerebrovascular interventions, and aid multidisciplinary decision-making in complex neurovascular cases. Validation in clinical practice is warranted to establish its utility.

## Introduction

Persistent primitive hypoglossal artery (PPHA) is an uncommon embryological remnant of fetal carotid-vertebrobasilar anastomoses. It arises from the cervical segment of the internal carotid artery (ICA), typically between C1 and C3, and enters the cranium through the hypoglossal canal to join the basilar artery [42]. PPHA results from the failure of regression of the embryonic hypoglossal artery during normal vascular development. While most often detected incidentally on cerebral imaging studies, its presence carries significant clinical and surgical implications, particularly in patients undergoing carotid artery interventions [10, 61].

The estimated incidence of PPHA ranges from 0.02% to 0.26%, making it the second most common persistent carotid-vertebrobasilar anastomosis after the persistent trigeminal artery. The presence of PPHA is often associated with ipsilateral hypoplasia or absence of the vertebral artery and posterior communicating artery, placing hemodynamic reliance on the ICA to supply both anterior and posterior circulation territories [66].

**Fig. 1:**
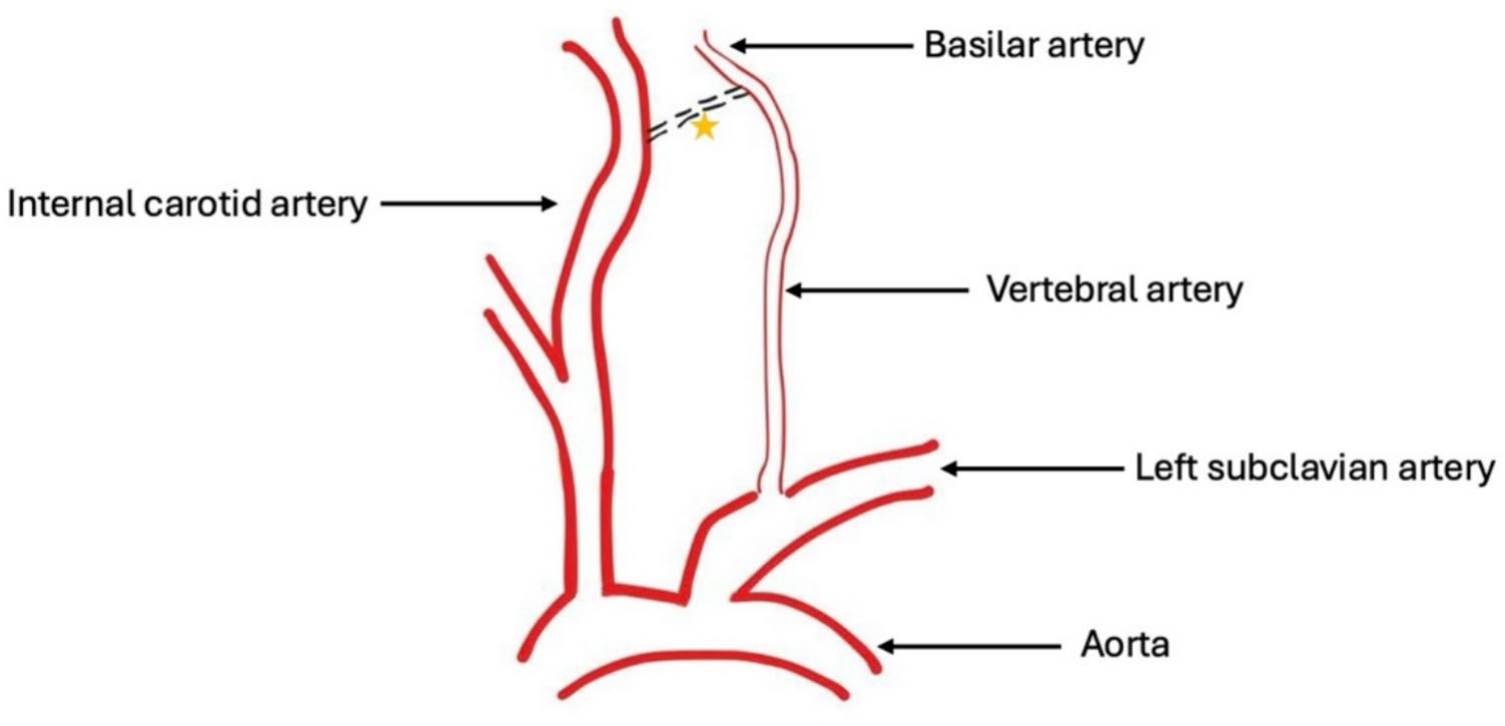
The normal regression of a PPHA between the basilar artery and the ICA. (Orange star denotes the rudimentary vessel of PPHA)

**Fig. 2:**
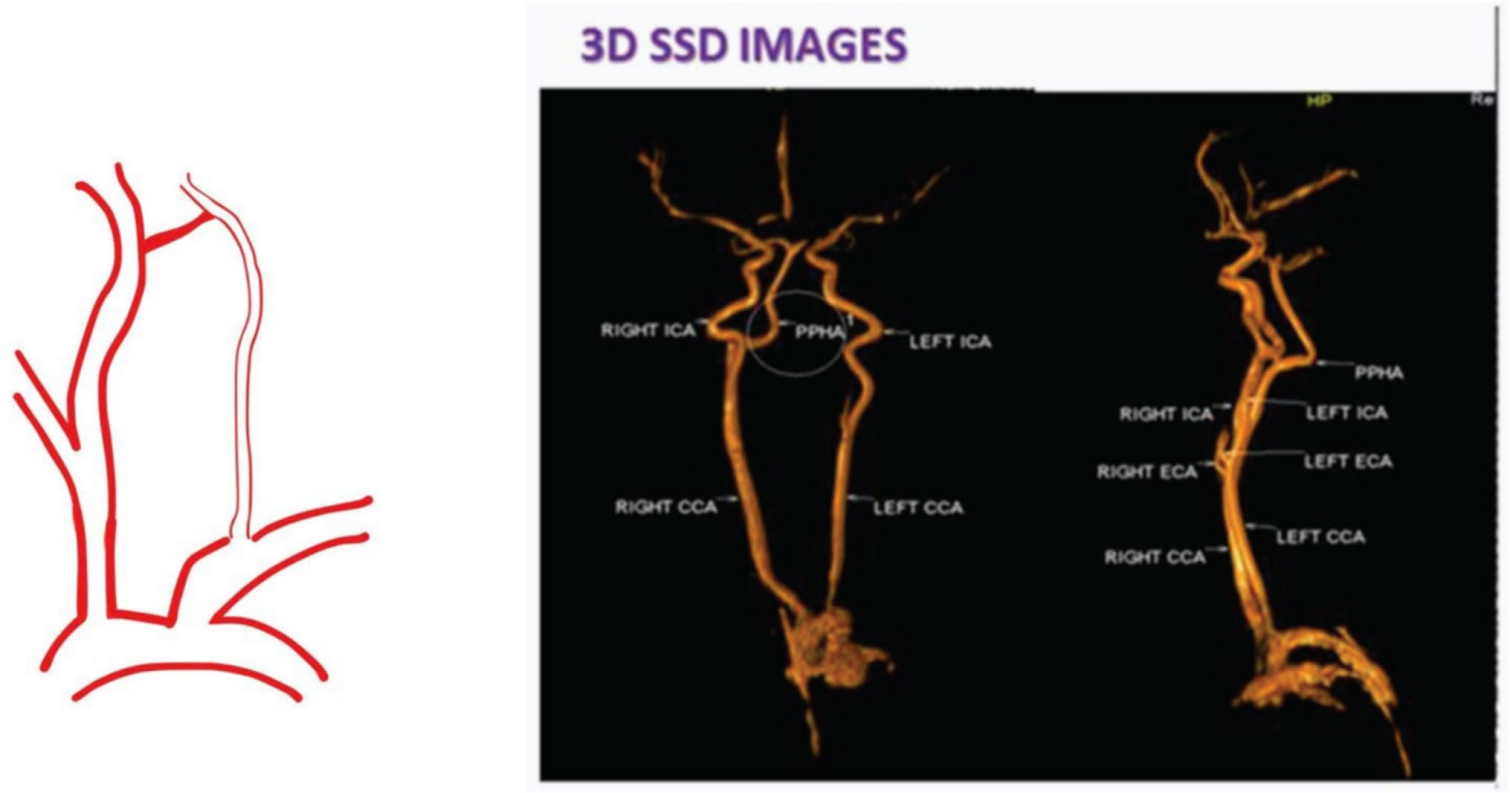
Category I PPHA: isolated asymptomatic PPHA. 3D Surface Shaded Display (SSD) showing PPHA with absent bilateral vertebral arteries [55]. Reproduced from Srinivas MR et al., J Clin Diagn Res. 2016;10(1):TD13–TD14. Used under the Creative Commons Attribution-NonCommercial (CC BY-NC) License. No changes were made

**Fig. 3:**
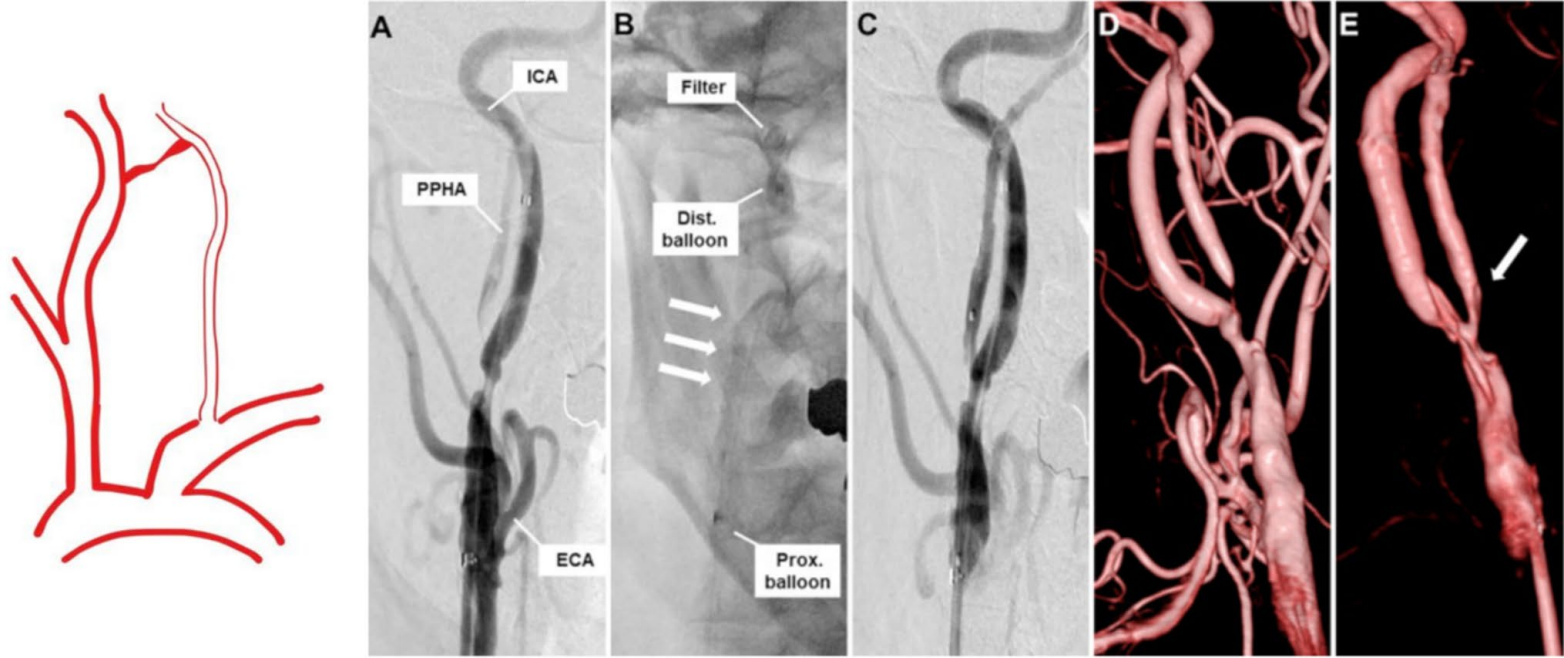
Category II PPHA: stenotic PPHA. A catheter-based angiogram was performed during percutaneous transluminal angioplasty (PTA) for a persistent primitive hypoglossal artery (PPHA). The pre-procedural anteroposterior image of the right common carotid artery (CCA) showed significant stenosis at the PPHA origin with markedly reduced flow. During PTA, a proximal balloon was positioned at the internal carotid artery (ICA) bifurcation, a distal balloon in the distal ICA, and a filter wire in the distal PPHA; angioplasty was then conducted using a 3.5 mm balloon. Post-angioplasty imaging demonstrated improved luminal caliber and enhanced flow through the PPHA. Three-dimensional digital subtraction angiography (3D-DSA) confirmed resolution of the stenosis after the procedure [24]. Reproduced from Iwaki K et al., J Neurosurg Case Lessons. 2023;6(17):CASE23427. Used under the Creative Commons Attribution-NonCommercial-NoDerivatives (CC BY-NC-ND) License. No modifications were made

**Fig. 4:**
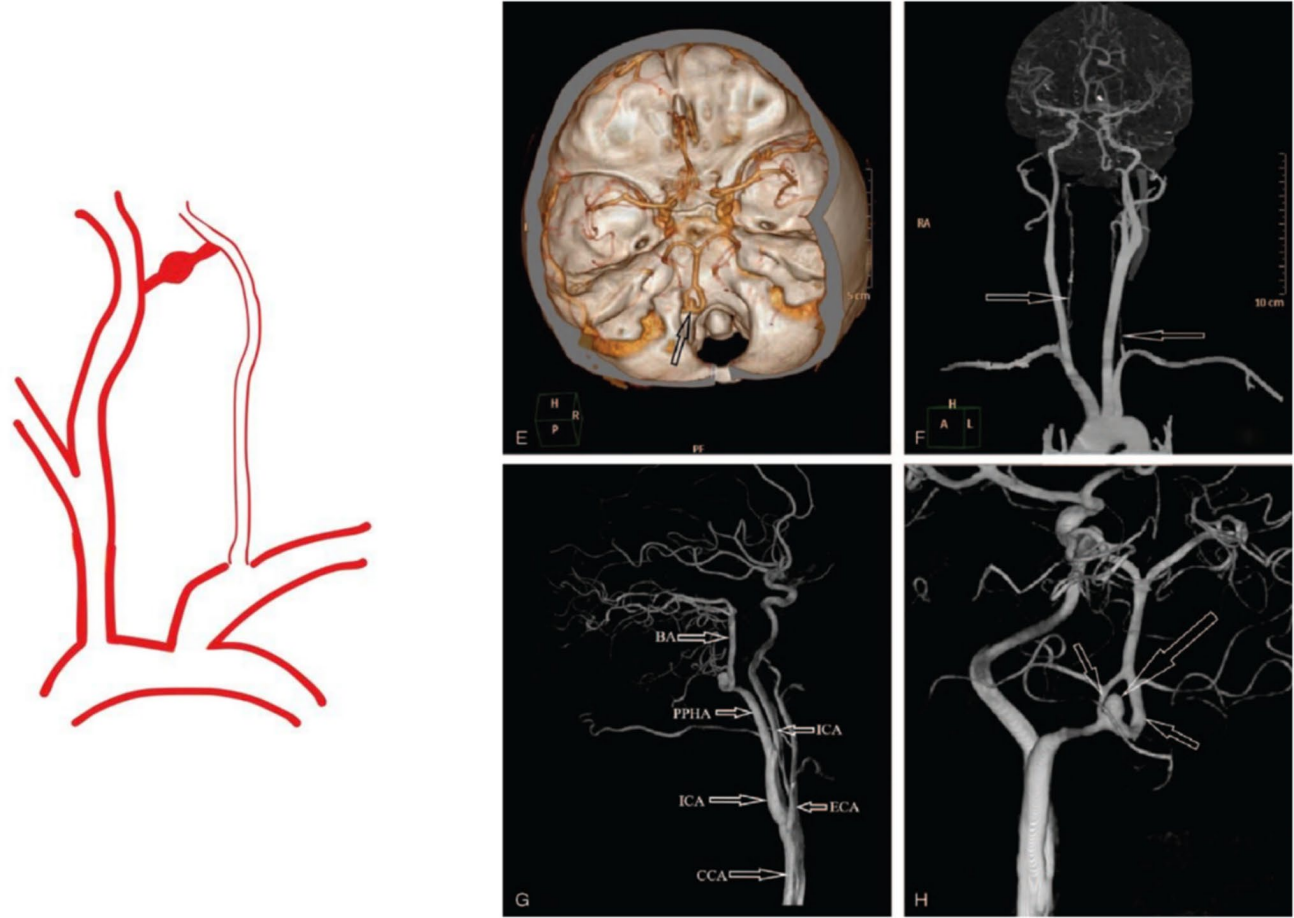
Category III PPHA: PPHA with aneurysm formation. Preoperative imaging (**A**–**D)** demonstrated intracranial hemorrhage and a persistent primitive hypoglossal artery (PPHA). Image **A** shows a CT scan revealing hemorrhage within the fourth ventricle, while image **B** demonstrates third and bilateral lateral ventricular hemorrhages. Image **C** identifies a PPHA arising from the internal carotid artery (ICA) at the level of the C1–C2 vertebrae, and image **D** shows its intracranial entry via the hypoglossal canal. Additional imaging (E–H) using CTA and DSA revealed a fenestrated vascular malformation with a cystic protrusion on the left side. Specifically, image **E** shows a fenestrated PPHA with a ruptured aneurysm and absent posterior communicating arteries; images **F**–**H** confirm hypoplastic vertebral arteries with no contribution to the basilar artery and a PPHA supplying the posterior circulation, including an associated aneurysm and vascular fenestration [17]. Reproduced from He S et al., Medicine (Baltimore). 2021;100(32):e26904. Used under the Creative Commons Attribution (CC BY 4.0) License

**Fig. 5:**
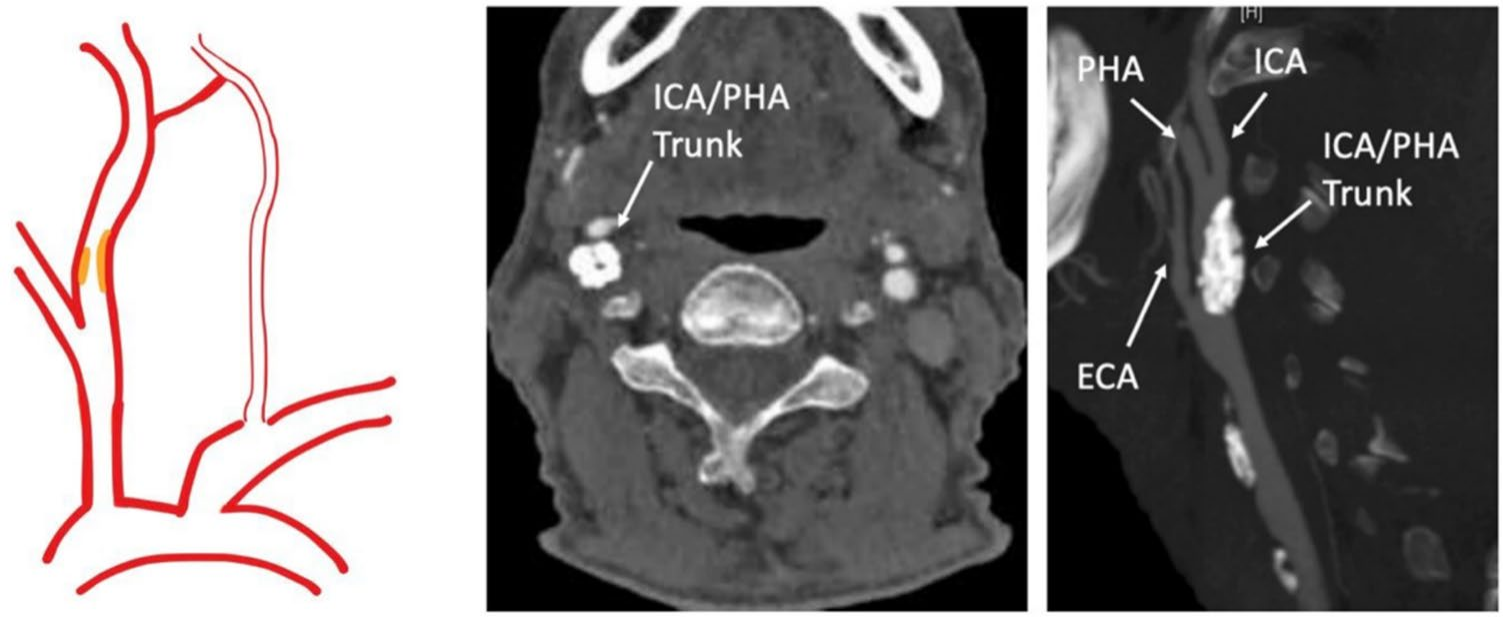
Category IV PPHA: PPHA with associated ipsilateral ICA stenosis. Sagittal image of the stenosis in which plaque extended from the common carotid artery (CCA) into the common ICA/PHA trunk, to just proximal to the ICA/PHA bifurcation. ECA, external carotid artery; ICA, internal carotid artery; PHA, persistent hypoglossal artery [37]. Reproduced from Leonard SD et al., J Vasc Surg Cases Innov Tech. 2024;11(1):101,648. Used under the Creative Commons Attribution-NonCommercial-NoDerivatives (CC BY-NC-ND 4.0) License. No modifications were made

**Fig. 6:**
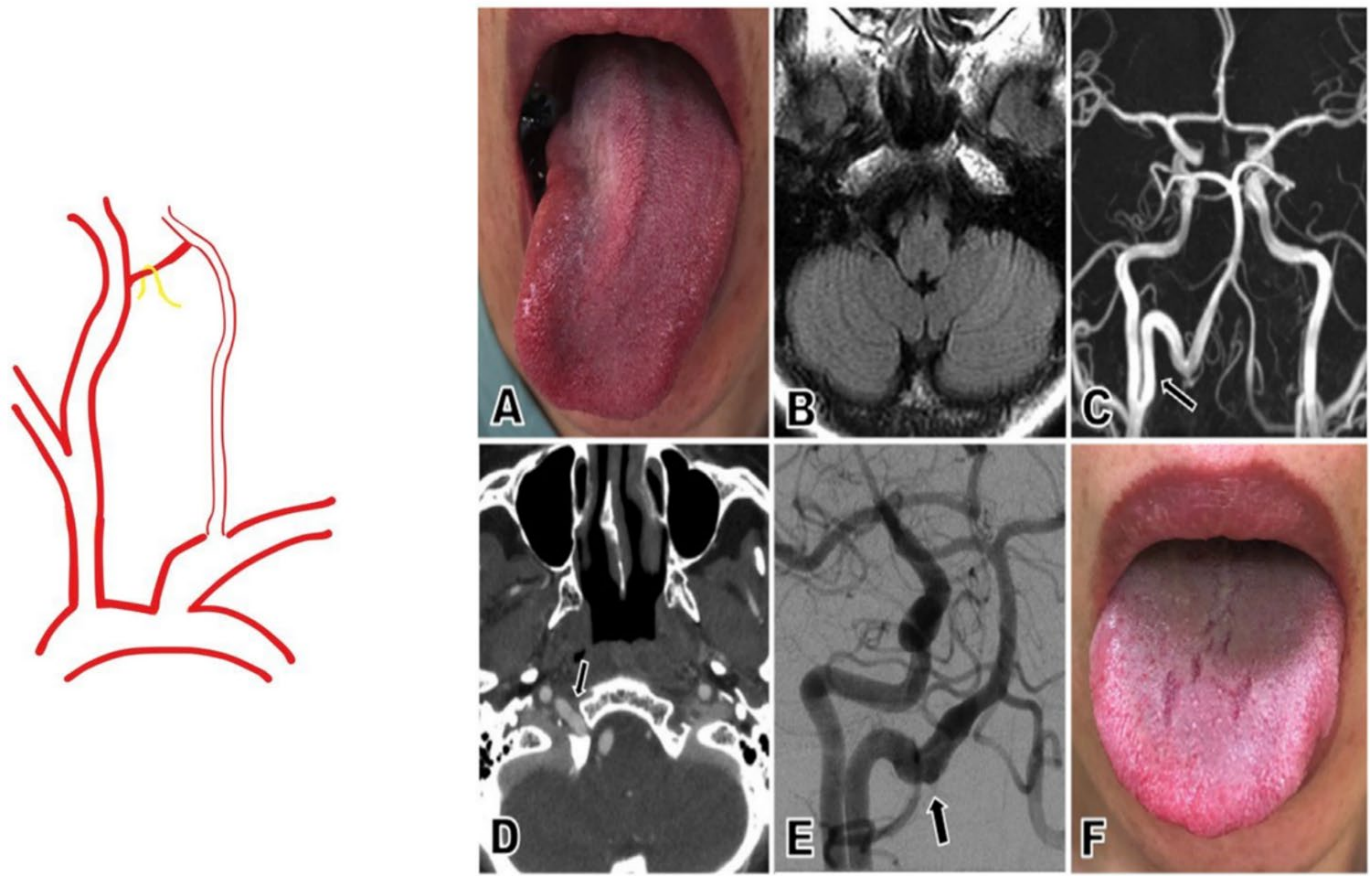
Category V PPHA: PPHA with cranial nerve compression. (**A**) Upon admission, neurological examination revealed rightward deviation of the patient’s tongue. (**B**) FLAIR imaging showed no evidence of pathology in the medulla oblongata. (**C**) Magnetic resonance angiography identified an unusual vessel arising from the right internal carotid artery (arrow). (**D**) CT angiography demonstrated this artery entering the skull via the right hypoglossal canal (arrow). (**E**) Digital subtraction angiography revealed an elongated persistent primitive hypoglossal artery with an irregular lumen within the hypoglossal canal (arrow). (**F**) The patient's right hypoglossal nerve palsy showed improvement one year after symptom onset [18]. Reproduced with permission from Hikichi et al., J Stroke Cerebrovasc Dis. 2020;29(2):104,459. © 2020 Elsevier. All rights reserved

**Fig. 7:**
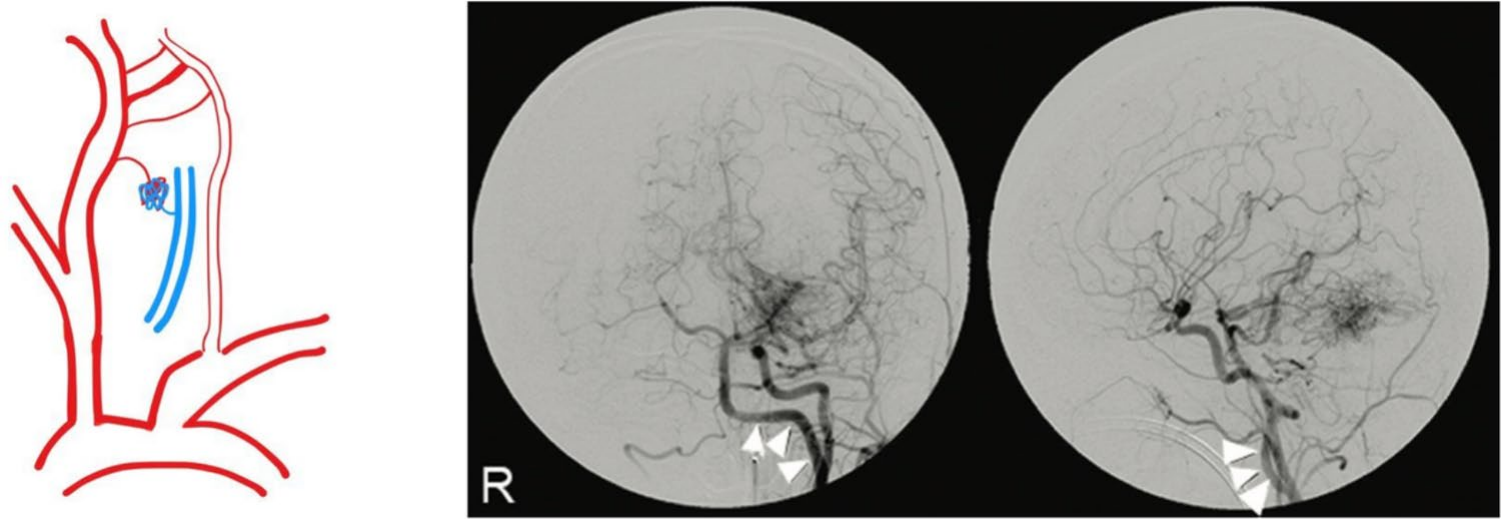
Category VI PPHA: PPHA with other associated vascular anomalies. Digital subtraction angiography of the left common carotid artery—shown in anteroposterior (left) and lateral (right) views—demonstrates an arteriovenous malformation nidus located in the left cerebellar hemisphere, along with visualization of a left-sided persistent primitive hypoglossal artery (PPHA; indicated by arrowheads).[27] Reproduced from Kageyama H et al., Surg Neurol Int. 2015;6:71. Used under the Creative Commons Attribution (CC BY 4.0) License

Recognition of PPHA is important not only for avoiding iatrogenic complications during endarterectomy or stenting but also for identifying patients at increased risk of ischemic or hemorrhagic complications due to associated vascular pathology. Despite its significance, the literature lacks a systematic classification scheme for PPHA. This manuscript presents a comprehensive six-category clinical classification intended to organise the anatomic and clinical diversity of PPHA and guide management [6] (Figs. 1, 2, 3, 4, 5, 6 and 7).

## Embryological context

In early embryonic life, multiple transient anastomoses form between the primitive internal carotid arteries and the paired longitudinal neural arteries, precursors of the basilar artery [36]. These include the trigeminal, otic, hypoglossal, and proatlantal arteries. As the vertebral arteries develop from the cervical intersegmental arteries and fuse to form the basilar artery, these primitive connections typically regress. However, when the hypoglossal artery persists into postnatal life, it becomes the PPHA. This artery maintains a direct connection between the ICA and the basilar artery, bypassing the vertebral arteries entirely or partially [14, 55].

The hypoglossal artery typically passes through the hypoglossal canal, closely associated with the hypoglossal nerve. Its persistence in adults often correlates with hypoplastic vertebral arteries and may have implications for cranial nerve compression, as well as for cerebral perfusion under conditions of carotid stenosis [25].

## Clinical classification of PPHA

### Category I: Isolated, Asymptomatic PPHA

This category includes patients in whom PPHA is found incidentally, often during angiography or cross-sectional imaging for unrelated conditions such as trauma or headaches [19]. Anatomically, the artery follows the classic course—arising from the ICA, coursing through the hypoglossal canal, and terminating in the basilar artery. In many cases, the ipsilateral vertebral artery and posterior communicating artery are absent or hypoplastic, making PPHA the principal posterior circulation supplier.

These patients are typically asymptomatic and exhibit no evidence of aneurysm or stenosis. While no immediate intervention is required, awareness of this configuration is essential for surgical planning to avoid posterior circulation compromise. Some authors recommend baseline vascular imaging and long-term follow-up in young or high-risk individuals [54]. An example of an isolated asymptomatic PPHA previously published under a Creative Commons license is shown below.

### Category II: PPHA with Stenosis

In this group, patients exhibit significant narrowing of the PPHA, usually at its origin or within the hypoglossal canal. Atherosclerotic disease is the most common aetiology, though fibromuscular dysplasia and other intrinsic vascular disorders may also contribute. As PPHA may be the primary source of blood to the posterior circulation, its stenosis can lead to vertebrobasilar insufficiency.

Patients may report dizziness, vertigo, blurred vision, syncope, or transient ischemic attacks. In advanced cases, brainstem infarction may occur. CT or MR angiography are useful for diagnosis, although digital subtraction angiography offers superior resolution.

Management includes antiplatelet therapy and control of modifiable risk factors. In symptomatic or high-grade stenosis, endovascular stenting or angioplasty may be indicated. Operators must proceed cautiously to avoid embolisation or vessel injury [48]. The following previously published case illustrates PPHA stenosis and its endovascular treatment, reused under appropriate license.

### Category III: PPHA with Aneurysm

This category describes cases in which an aneurysm is identified along the PPHA or at its junction with the basilar artery. Hemodynamic stress due to anomalous flow dynamics, vessel wall weakness, and segmental dysplasia is considered a contributing factor.

Patients may remain asymptomatic until aneurysm rupture, which presents with acute subarachnoid haemorrhage. Some may exhibit warning signs such as severe headaches or cranial neuropathies. In cases where the aneurysm is unruptured but deemed at high risk, prophylactic treatment may be considered.

Treatment strategies include endovascular coiling, balloon-assisted coiling, or stent-assisted techniques. Surgical clipping is generally reserved for anatomically unsuitable cases. Outcomes are influenced by aneurysm location, size, and rupture status at diagnosis [4, 8, 60]. A previously published case demonstrating PPHA-associated aneurysm formation is included for reference.

### Category IV: PPHA with Ipsilateral Carotid Artery Stenosis

In patients where PPHA coexists with significant ipsilateral ICA stenosis, cerebral perfusion becomes critically dependent on this single conduit. During carotid revascularisation, inadvertent interruption of flow can result in bilateral hemispheric and posterior circulation ischemia [35].

Clinical presentation may include features of both anterior and posterior circulation compromise: transient hemiparesis, aphasia, visual field deficits, diplopia, or ataxia. Accurate identification is essential during preoperative planning for carotid endarterectomy (CEA) or stenting (CAS).

CEA in such cases requires specialised techniques such as dual shunting to maintain perfusion. CAS may necessitate double embolic protection devices. Procedural planning should be multidisciplinary and individualised [29, 37]. A previously published case illustrating PPHA with ipsilateral ICA stenosis is presented below, reproduced with permission.

### Category V: PPHA with Cranial Nerve Compression

Due to its proximity to the hypoglossal nerve, an enlarged or tortuous PPHA can compress adjacent neural structures, most commonly cranial nerve (CN) XII. Symptoms include unilateral tongue weakness, dysarthria, dysphagia, and in some cases, chronic pain or sensory deficits.

Imaging may show expansion of the hypoglossal canal and displacement of the nerve by the pulsatile vessel. Conservative management with observation and medical therapy may suffice in mild cases. Progressive symptoms may require vascular decompression [18, 41]. An example of cranial nerve XII compression by PPHA is illustrated below in a previously published case, with permission for reuse.

### Category VI: PPHA with Associated Vascular Anomalies

PPHA may coexist with other vascular abnormalities, including persistent trigeminal or proatlantal arteries, arteriovenous malformations (AVMs), Chiari malformations, and aneurysms at other intracranial locations. These complex variants can result in unusual hemodynamic patterns and may pose challenges in both diagnosis and intervention.

Clinical manifestations vary widely but may include seizures, chronic headaches, or focal neurologic deficits. A high index of suspicion is warranted, and comprehensive multimodal imaging should be employed. Treatment is tailored to the specific anomalies present and may require a multidisciplinary approach involving neurology, neurosurgery, and endovascular specialists [15, 47, 57, 67]. A previously published case demonstrating PPHA with an associated AVM is presented here.

A summary of the different categories, including their clinical features and therapeutic approaches, can be found in Table 1.

**Table 1:** Summary of PPHA Classification

Category	Description	Clinical Features	Management
I	Isolated, asymptomatic PPHA	Incidental finding	Observation
II	PPHA with stenosis	Dizziness, TIAs, vertigo	Medical therapy; Angioplasty ± stenting
III	PPHA with aneurysm	Headache, SAH	Endovascular or surgical repair
IV	PPHA with ICA stenosis	Mixed anterior/posterior symptoms	CEA with shunting or protected CAS
V	Cranial nerve (CN) compression	CN XII palsy	Conservative; Decompression surgery
VI	Associated vascular anomalies	Variable (AVM, aneurysm, Chiari)	Multidisciplinary approach

*AVM* = Arteriovenous Malformation, *CAS* = Carotid Artery Stenting, *CEA* = Carotid endarterectomy, *CN* = Cranial Nerve, *ICA* = Internal Carotid Artery, *PPHA* = Persistent primitive hypoglossal artery, *TIA* = Transient Ischemic Attack, *SAH* = Subarachnoid haemorrhage

## Discussion

This proposed six-tier clinical classification organises PPHA into discrete categories based on anatomical complexity, associated vascular pathology, and clinical presentation. This framework is designed to support decision-making in both elective and emergent clinical settings. Importantly, it underscores the need for careful preoperative evaluation of patients undergoing carotid interventions [20, 29].

A comprehensive search of PubMed, Scopus, and Google Scholar was performed for all publications from database inception to June 2025 using the terms “persistent primitive hypoglossal artery” and “PPHA”. References from identified articles were also screened for additional relevant cases. Cases were then categorised according to the proposed six-tier PPHA classification. A brief literature review of the most important published studies on cases of PPHA, their presentations and corresponding treatment are demonstrated in Table 2.

**Table 2:** Brief literature review of different pathologies, presentations, and corresponding treatment strategies used in different clinical scenarios associated with PPHA

Published literature	Pathology	Presentation	Treatment
Yoshida S, et al. (2023)[65]	Severe cervical ICA stenosis proximal to origin of PPHA	Vertebrobasilar insufficiency (posterior circulation symptoms)	CEA or CAS reported across cases & PTA of PPHA origin
Yamamoto S, et al. (1991) [64]	Ruptured aneurysms of PPHA	SAH	Open microsurgical clipping
Zhang L, et al. (2021) [67]	Basilar bifurcation aneurysm adjacent to PPHA	SAH	Y-stent assisted coil embolization of basilar bifurcation aneurysm
AlQarni AA, et al. (2024) [1]	Isolated PPHA visualized	Syncope	Conservative/imaging surveillance
Ryu B, et al. (2016) [50] Murai S, et al. (2016) [43]	Cervical ICA stenosis with ipsilateral PPHA	Asymptomatic & symptomatic carotid disease	CAS
Hikichi H, et al. (2020) [18] Meila D, et al. (2012) [41]	Ectatic/calcified PPHA compressing hypoglossal canal/nerve	Dysarthria, tongue weakness (hypoglossal nerve palsy)	Management variation (conservative, neurosurgical)
Wan Z, et al. (2022) [63]	PPHA with multiple aneurysms (aneurysms on basilar bifurcation, PICA, AchoA)	SAH, incidental finding	Endovascular coil/stent techniques & open surgery
Paraskevas GK, et al. (2007) [46]	PPHA anastomosis visualized at autopsy	None	None

*AchoA* = Anterior Choroidal Artery, *CAS* = Carotid Artery Stenting, *CEA* = Carotid Endarterectomy, *ICA* = Internal Carotid Artery, *SAH* = Subarachnoid Hemorrhage, *PICA* = Posterior Inferior Cerebellar Artery, *PPHA* = Persistent Primitive Hypoglossal Artery, *PTA* = Percutaneous Transluminal Angioplasty

Based on the previously published studies, the number of cases reported in the previous literature is close to 60 patients and is summarised according to the introduced classification system, as seen in Table 3.

**Table 3:** Number of patients reported for each category of the introduced classification system

Category	Verified no. patients	Key references	
I. Isolated, asymptomatic PPHA	Unknown (under-reported)	Guzman R, et al. (2005) [13] Hopf-Jensen S, et al. (2017) [19]	Paraskevas GK, et al. (2007) [46] Avcu S, et al. (2009) [2]
II. PPHA with primary PPHA stenosis	3	Iwaki K, et al. (2023) [24] Sunada I, et al. (1991) [56]	Touho H, et al. (1994) [59]
III. PPHA with aneurysm	24	Hui FK, et al. (2011) [21] Kanai H, et al. (1992) [28] Baltsavias GM, et al. (2006) [3] Grand M, et al. (2005) [11]	De Blasi R, et al. (2009) [5] Kimball D, et al. (2015) [33] Murayama Y, et al. (1985) [44] Varvari I, et al. (2018) [62]
IV. PPHA with ICA stenosis	19	Kawamura K, et al. (2021) [32] Pride LB, et al. (2020) [49] Ryu B, et al. (2016) [50] Yoshida S, et al. (2023) [65] Shchanitsyn IN, et al. (2021) [53] Murai S, et al. (2016) [43] Telianidis S, et al. (2023) [58] Ishizuka T, et al. (2024) [23] Kanazawa R, et al. (2008) [29]	Kadooka K, et al. (2025) [26] Sanada T, et al. (2021) [51] Segawa M, et al. (2022) [52] Burgard M, et al. (2021) [7] Zhang L, et al. (2016) [68] Yuasa H, et al. (2005) [66] Megyesi JF, et al. (1997) [40] Kawabori M, et al. (2009) [31] Katoh M, et al. (1999) [30]
V. Cranial nerve compression	2	Hikichi H, et al. (2020) [18]	Meila D, et al. (2012) [41]
VI. Associated vascular/structural anomalies	9	Fujii Y, et al. (1988) [9] Hatayama T, et al. (1999) [16] Huynh-Le P, et al. (2004) [22] Kobayashi M, et al. (2008) [34]	Gupta M, et al. (2010) [12] Matsumura M, et al. (1985) [39] Nagarajan K, et al. (2025) [45]

In patients with PPHA and concurrent ICA disease, unrecognised vascular anatomy may result in devastating ischemic complications. Similarly, failure to recognise a PPHA-associated aneurysm may delay lifesaving interventions. Moreover, the presence of PPHA may necessitate tailored imaging protocols and individualised management strategies [38, 62].

Although PPHA is rare, its presence often coincides with high-risk vascular pathologies, where unrecognised anatomy can lead to devastating outcomes. The integration of quantified literature data into the proposed classification enhances its practical utility, enabling clinicians to anticipate procedural challenges, optimise perioperative planning, and improve patient safety when this anomaly is encountered.

The classification supports a holistic approach to management; one that prioritises anatomical assessment, individualised treatment planning, and cerebral protection. In patients undergoing carotid interventions, unrecognised PPHA can lead to severe complications, especially when it serves as the primary conduit for the posterior circulation. Tailored imaging, multidisciplinary collaboration, and procedural planning are essential to minimise risk [17, 49].

While no classification can encompass every anatomical variant, the proposed system offers a structured foundation for risk evaluation and clinical decision-making. As the first structured, clinically oriented classification of PPHA, it aims to support both diagnostic clarity and therapeutic planning. Its adoption in clinical practice may promote the development of registries and prospective studies, which are essential to validate its utility, refine risk stratification, and optimise patient outcomes.

## Conclusion

Persistent primitive hypoglossal artery represents a rare but clinically significant vascular anomaly. While often incidental, its presence can have profound implications when associated with vascular pathology. The classification system presented here offers a pragmatic framework for diagnosis, risk stratification, and procedural planning. It also enhances clarity in describing complex vascular anatomy and improves communication among physicians when discussing pathology or planning management. Future multicenter studies are needed to validate its utility and impact on outcomes.

## Data Availability

No datasets were generated or analysed during the current study.

## Abbreviations:

AVM: Arteriovenous Malformation
ICA: Internal Carotid Artery
PPHA: Persistent Primitive Hypoglossal Artery
CAS: Carotid Artery Stenting
CEA: Carotid endarterectomy
CN: Cranial Nerve
ICA: Internal Carotid Artery
PPHA: Persistent primitive hypoglossal artery
TIA: Transient Ischemic Attack
SAH: Subarachnoid haemorrhage

## Declarations

### Additional information

The local institutional review board (IRB) approved the current review study (R.25.05.3183–2025/05/14).
